# Statistical Object Data Analysis of Taxonomic Trees from Human Microbiome Data

**DOI:** 10.1371/journal.pone.0048996

**Published:** 2012-11-09

**Authors:** Patricio S. La Rosa, Berkley Shands, Elena Deych, Yanjiao Zhou, Erica Sodergren, George Weinstock, William D. Shannon

**Affiliations:** 1 Division of General Medical Sciences, Washington University in St. Louis, St. Louis, Missouri, United States of America; 2 The Genome Institute, Washington University in St. Louis Medical School, St. Louis, Missouri, United States of America; National Taiwan University, Taiwan

## Abstract

Human microbiome research characterizes the microbial content of samples from human habitats to learn how interactions between bacteria and their host might impact human health. In this work a novel parametric statistical inference method based on object-oriented data analysis (OODA) for analyzing HMP data is proposed. OODA is an emerging area of statistical inference where the goal is to apply statistical methods to objects such as functions, images, and graphs or trees. The data objects that pertain to this work are taxonomic trees of bacteria built from analysis of 16S rRNA gene sequences (e.g. using RDP); there is one such object for each biological sample analyzed. Our goal is to model and formally compare a set of trees. The contribution of our work is threefold: first, a weighted tree structure to analyze RDP data is introduced; second, using a probability measure to model a set of taxonomic trees, we introduce an approximate MLE procedure for estimating model parameters and we derive LRT statistics for comparing the distributions of two metagenomic populations; and third the Jumpstart HMP data is analyzed using the proposed model providing novel insights and future directions of analysis.

## Introduction

The Human Microbiome Project (HMP) [1] was initiated by the NIH to identify and characterize the microbes and their communities found in or on human body, focusing on the nasal cavity, oral cavity, vagina, skin, and gastrointestinal tract. The goal of the HMP is “determining whether individuals share a core human microbiome, understanding whether changes in the human microbiome can be correlated with changes in human health, and developing the new technological and bioinformatics tools needed to support these goals” [2].

Microbiome samples are collected from patient Body sites by swabbing (e.g., skin, nasal, oral) or bulk collection (e.g., saliva, stool). These samples contain within them the entire bacterial community (i.e., the microbiome) as well as other organisms (e.g., human cells, viruses, fungi). Samples are processed to isolate the genomic content (i.e., all DNA from the entire bacterial microbiome, all patient DNA, all viral DNA, etc.) within that sample, and prepared for state-of-the-art ‘next generation’ sequencing. To characterize the microbial community structure, 16S rRNA genes are sequenced using the high throughput 454 FLX Titanium sequencing platform (Roche). The sequences are analyzed using either a phylogenetic or taxonomic approach [3], [4]. The phylogenetic approach studies communities’ evolutionary relationships between sequences within the sample, and generally represents the microbiome by a phylogenetic tree. The taxonomic approach assigns sequences to taxonomic units using unsupervised and supervised methods: The unsupervised taxonomic method computes pairwise nucleotide distances between 16S rRNA gene sequences and an operational taxonomic unit (OTU) is assigned, by alignment-based clustering, to sequences that are at least 97% identical; and the supervised taxonomic method matches a sequence to a hierarchical taxa or taxonomy bins defined in a bacterial-taxonomy library such as, for example, the Ribosomal Database Project (RDP) [5], Greengenes [6], SILVA [7], and GAST [8]. The supervised taxonomic analysis allows us to represent each sample (set of sequences) by a rooted taxonomic tree where the root corresponds to taxon at the Kingdom level, i.e., bacteria, and the leaves correspond to the taxa at the Genus level, and the width of the edges (paths) between taxonomic levels correspond to the abundances of the descending taxon. A number of reviews on the phylogenetic and taxonomic analysis of sequences have appeared recently (e.g. see [9], [10], [4]). In [4], the authors showed that both the supervised and unsupervised taxonomic methods arrive at similar ecological/biological conclusions. However, the supervised taxonomic analysis is more tolerant to sequencing errors, and it requires significantly less computational power than the taxonomy unsupervised analysis [4].

With the goal to enumerate the content and abundances in the microbial communities of 18/15 body habitats of 300 healthy female/male adults, 7,000 16S rRNA sequences were produced from an individual on average per body site sample. These sequence data sets provide the opportunity to estimate the microbial diversity with high resolution, but statistical tools and strategies to analyze the microbial communities are needed to take full advantage of the data density.

In recent years several tools have been developed to compare Human microbiome communities using either phylogenetic or taxonomical classification of metagenomic sequences. Current strategies are based primarily on exploratory cluster analysis, phylogenetic inferences, biological diversity indices, bootstrap or resampling methods, and application of univariate and non-parametric statistics to different subsets of the data [11]–[23].

Tools currently being used to analyze HMP data for limited numbers of sequence reads include UniFrac [14], SONS [12], and DOTUR [11]. UniFrac, for example, uses phylogenetic distances and permutation testing to compare samples, which does not require a large number of sequence reads to detect significant differences between two samples. SONS and DOTURS compare similarities between samples using OTU-based taxonomies and standard diversity indices of complexity, which provide with quantitative descriptions of a community as well as of its similarity to other communities. Several other methods exist that depend on sequence and phylogeny comparisons (e.g., AMOVA, Tree Climber) [15] or diversity indices and community coverage (e.g., LIBSHUFF [16] and S-LIBSHUFF [17]). All the above methods compare two libraries of sequences, however, because of the computational complexity of calculating phylogenetic trees and generating huge pairwise distance matrices between sequences, these methods are meant mainly to perform pairwise sample comparisons of at most two groups of samples with a restricted amount of sequences per samples. Analysis of groups of large HMP samples has been limited to the application of clustering and ordination techniques based on pairwise distances between samples (see for example, [24]). Other methods and ecological/HMP software packages compare microbiomes based on standard statistical methods such as contingency tables, Fisher’s Exact Test, or goodness-of-fit tests to multinomial distributions, bootstrap tests. These packages (e.g., ANOSIM [25], XIPE-TOTEC [18], IMG/M [19], MEGAN [20], Metastats [21], QIIME [22], and STAMP [23] require a significant reduction of the HMP data, often basing the statistical analyses on pairwise comparisons of the abundances of taxa bin or OTUs or other summary statistic features (e.g., community functions).

In this work a novel parametric statistical inference method based on object-oriented data analysis (OODA) for analyzing HMP data is proposed. OODA is an emerging area of statistical inference where the goal is to apply statistical methods to objects such as functions [26], images, and graphs or trees [27], [28]. In particular, the data objects that pertain to this work are RDP-based taxonomic trees. There is one such object for each habitat sites sampled from each person, and, thus we are interested in modeling and comparing population of trees. The probabilistic modeling approach proposed here has been applied previously to hierarchical clustering trees (dendrograms) and classification and regression trees [29], [30], [27], as well as for constructing Maximum Likelihood Supertrees [31]. This contribution is threefold: first, a weighted tree structure to analyze RDP data is introduced; Second, the unimodal probability measure proposed in [29], [27] is applied to model a set of RDP trees, and the likelihood ratio test statistics for comparing the probability models of two microbiome populations is derived; and third the HMP data is analyzed using the proposed model providing novel insights. An R-package has been developed containing the implementations of the visualization and methods proposed on this work [32].

**Table 1:** Example of a Linnaean taxonomic classification of three sequences.

Seq. ID	Kingdom	Phylum	Class	Order	Family	Genus
F51YIRY01BC31	Bacteria:0.99	Bacteroidetes:0.99	Bacteroidia:0.9	Bacteroidales:0.99	Prevotellaceae:0.99	Prevotella:0.99
F51YIRY01DFQI	Bacteria:0.99	Firmicutes:0.53	Clostridia:0.53	Clostridiales:0.53	Veillonellaceae:0.53	Megasphaera:0.52
F51YIRY01CLKP	Bacteria:0.99	Firmicutes:0.96	Bacilli:0.91	Lactobacillales:0.90	Enterococcaceae:0.44	Pilibacter:0.41

Each taxa assignment shows the estimated classification reliability computed via bootstrapping.

Of primary interest to HMP investigators is the estimation of the core microbiota from a set of samples. Determining a core microbiota aims at finding the organisms (or functions) selected in the host environment, and at studying its correlation with changes in human health. By defining a unimodal probability measure we are able to compute a central taxonomic tree, the maximum likelihood tree, providing an alternative and new definition of the core-microbiome for a set RDP trees samples. Though, in this paper we are focused on analyzing 454 sequencing of 16S rRNA genes with the reads mapped to taxonomic (classification) assignments, the methods are equally applicable to shotgun sequencing data with functional profiling of the microbial community.

**Figure 1:**
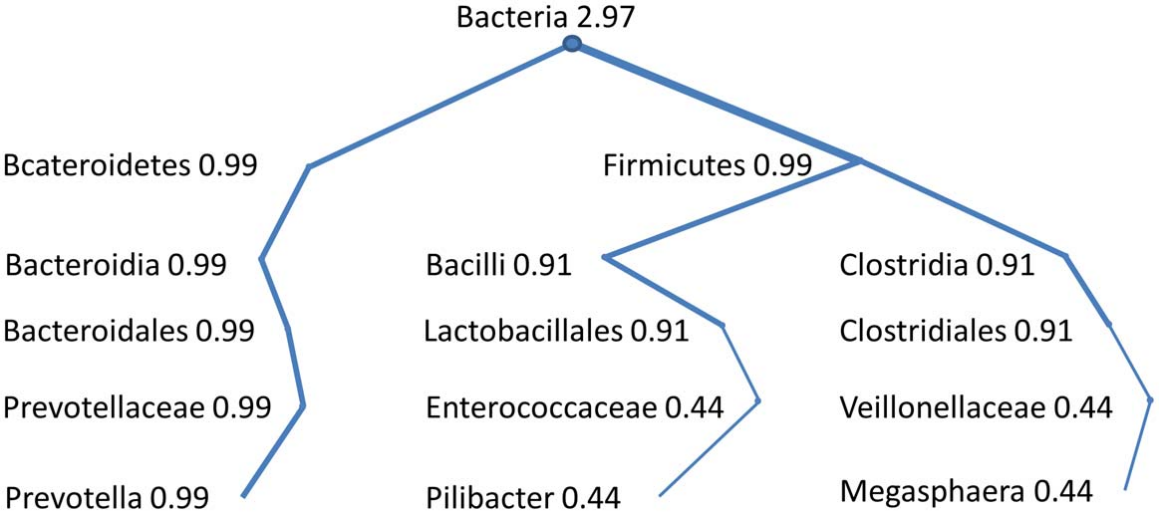
Example of a bacterial taxonomic tree build from adding three RDP classifications of sequences as shown in **Table 1**.

## Materials and Methods

### Ethics Statement

Subjects involved in the study provided written informed consent for screening, enrollment and specimen collection. The protocol was reviewed and approved by Institutional Review Board at Washington University in St. Louis. The data were analyzed without personal identifiers. Research was conducted according to the principles expressed in the Declaration of Helsinki. This manuscript adheres to the HMP data release policy (see [33] and [1] for more details).

**Figure 2:**
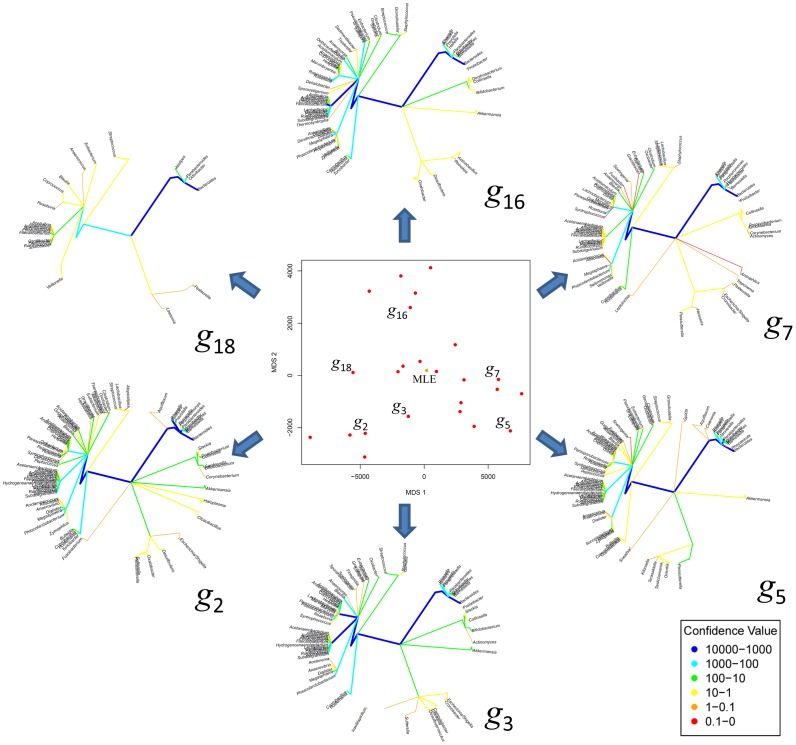
MDS plot showing the distribution of the taxonomic trees corresponding to stool samples sequenced at region V3–V5. The MLE tree of all samples is denoted by MLE (dot in black) in the MDS plot. Individual taxonomic trees are denoted by 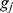 with 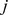 = {2, 3, 5, 7, 16, 18} and these are shown around the MDS plot to illustrate how the tree structure varies. The tree branches are color-coded to represent their weight values (sum of confidence) according to the reference table at the bottom left side of the plot. Blue denote the branches with the highest confidence among all while red denote the branches with lowest confidence. Note here that the tip of each branch represents a genus, and the location of each genus is the same on all trees.

**Figure 3:**
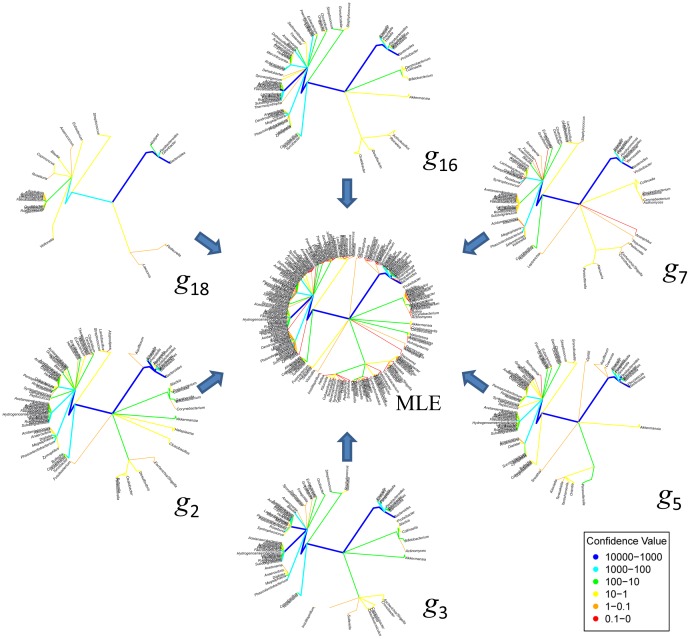
Illustration of the MLE tree for stool samples, region V3–V5. Sample individual taxonomic trees shown in Figure 2 (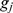 with 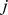 = {2, 3, 5, 7, 16, 18}) are displayed around the MLE tree to illustrate some of tree structures represented by the MLE tree.

### HMP Data Description and Data Structure

Human microbiome data analyzed in this paper for illustration purposes are from samples of 24 subjects (male and female), 18–40 years old, from two geographic regions of the US: Houston, TX and St. Louis, MO. These samples were collected as part of study HMP: 16S rRNA 454 Clinical Production Pilot (Project ID: 48335) (see [34]). Each sample was distributed to two HMP sequencing centers, and ∼1×105 sequences were obtained from two regions of the 16S ribosomal RNA gene, denoted as variable regions V1–V3 and V3–V5. The sequences were assigned to bacterial taxa by matching the DNA sequence reads to bacterial reference sequences using the RDP [5]. RDP matches each rRNA sequence to a set of hierarchical taxa following a Linnaean-based taxonomy, and it provides a confidence score, computed via bootstrapping, for each taxonomic classification [5]. The matching is done using a naïve Bayesian rRNA classifier which is trained on the known type strain 16S sequences (and a small number of other sequences representing regions of bacterial diversity with few named organisms) [5]. Generally as a read is assigned further down the taxonomy from kingdom to genus level there is less confidence since reads may show partial matching at more specific taxonomic levels, as well as matching to multiple taxa. This is illustrated in Table 1 where three sequence reads are mapped down to the genus level, with the associated confidence value at each level. Each sequence read’s RDP match defines a taxonomic tree path, and when combining them together forms a natural representation of the HMP sample as a Linnaean taxonomic tree. A taxonomic tree is an acyclic rooted graph in which any two vertices or taxa are connected by a path or edge. HMP data is naturally represented as a rooted taxonomic tree with higher taxonomic levels (e.g., phylum or class versus family or genus) closer to the root, and edges weighted by the RDP confidence score. So far the tendency to combine RDP matches consists of applying a hard threshold filter to the confidence scores, usually at 80% or at 50%, and then overlap all branches by adding the filtered confidence scores of common paths. In the above approach, taxa with confidence scores above or equal to the threshold are assigned a score of 1, and below it are reassigned to an unknown taxa category with a score of 1. The path weights of the taxonomic tree obtained after combining the set of RDP matches provides with a measure of the abundance for the descending taxa. The above approach is somehow arbitrary since taxa abundances of known and unknown taxon will depend on the specific threshold level used. In this work, we will combine RDP values without using a threshold filter, which allows us to provide a measure of taxa abundance weighting on the confidence of each taxa assignment, and to avoid creating arbitrarily unknown taxa at each taxonomic level. For the three sequence reads in Table 1, the tree in Figure 1 is formed by adding confidence values for reads with overlapping paths. In this example all three sequence reads contribute 0.99 confidences to the Kingdom Bacteria (0.99+0.99+0.99 = 2.97), while sequence 2 and 3 contribute to Phylum Firmicutes (0.53+0.96 = 1.49). Note that all taxonomic levels provide important information to characterize the sample since the aggregated confidence at a parent node in the tree is not necessarily equal to the addition of the aggregated confidences from their children nodes.

**Figure 4:**
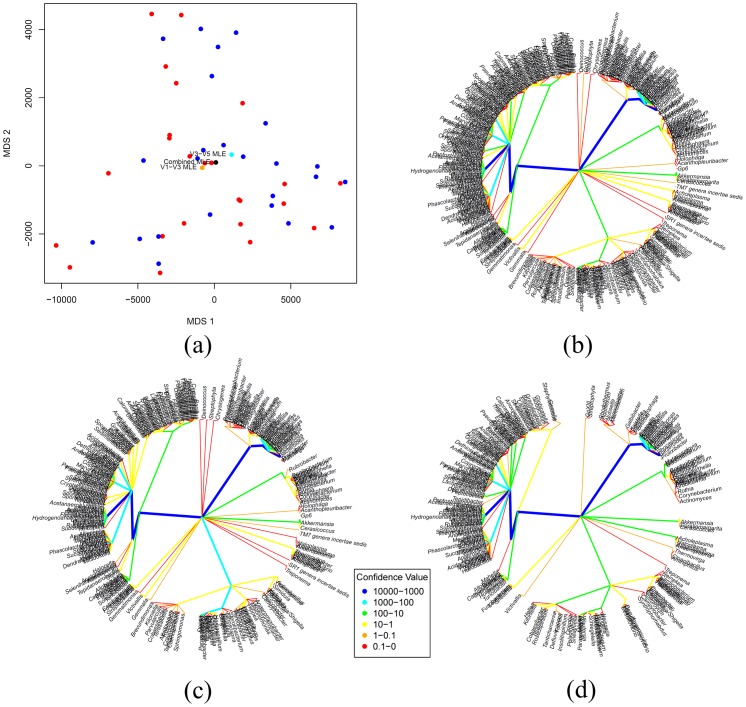
Analysis of stool samples for 24 subjects sequenced at variable regions V1–V3 and V3–V5, mapped to the RDP database. In Figure (a), a pairwise distance matrix was generated using Euclidean distance, and multidimensional scaling was used to display the distribution of these 48 trees showing V1–V3 (red) and V3–V5 (blue) samples are overlapping; In Figure (b), the MLE tree for the 48 trees is illustrated; and in Figures (c) and (d), the MLE tree for trees corresponding to V1–V3 and V3–V5 regions are shown, respectively.

**Table 2:** P-values of the two sample test comparison, using LRT statistics and 1000 bootstraps, to test for similarities across samples from variable regions V1–V3 and V3–V5 of the 16S rRNA gene, within a body site.

Body Habitats	P-value
anterior-nares	0.15
attached-gingivae	<E-03
buccal-mucosa	0.02
hard-palate	0.12
l-retroauricular-crease	0.47
mid-vagina	0.23
palatine-tonsils	0.03
posterior-fornix	0.22
r-retroauricular-crease	0.53
saliva	0.02
stool	0.26
subgingival-plaque	0.12
supragingival-plaque	0.12
throat	0.10
tongue-dorsum	0.05
vaginal-introitus	0.20

Building a taxonomic tree based on adding RDP confidences has several important properties. For example, the resulting tree is consistent with the RDP classification of each sequence where branches closer to the root have higher values than branches closer to leaves. Also, this approach provides with a linear approximation of the overall confidence of a branch in a sample, which allows us to identify tree branches that have overall higher confidence in each sample. Moreover, as stated above, for any given branch the addition of the confidence values provides with a measure of taxa abundance weighting on the confidence of the resulting RDP taxa assignment. However, one drawback of this approach is that Trees with larger number of sequence reads would tend to have branches with larger weight values, and thus would tend to bias the analysis when modeling a set of Trees, e.g., the computation of the MLE tree. Therefore, to avoid this issue in this work we normalize the number of sequence reads of all samples by a common number of reads.

**Figure 5:**
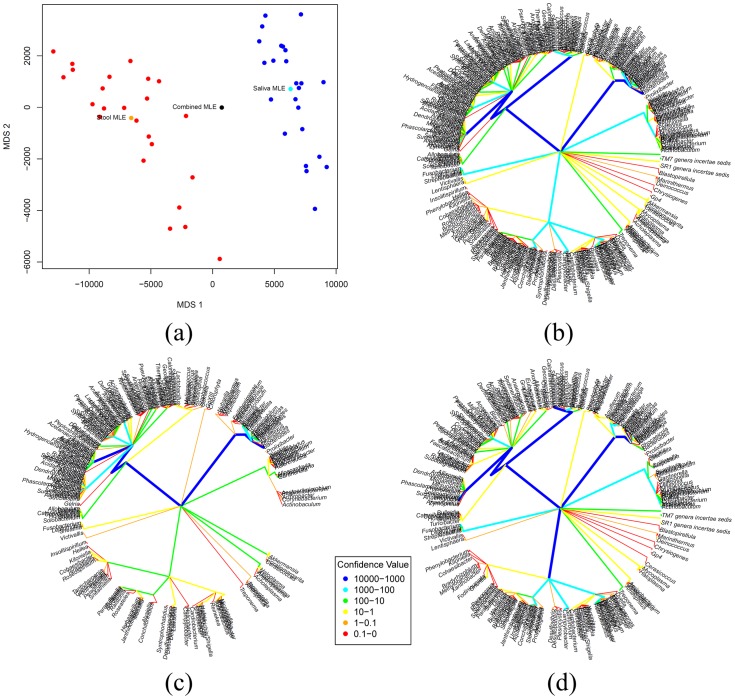
Analysis of saliva and stool samples for 24 subjects sequenced at variable regions V3–V5, mapped to the RDP database. In Figure (a), a pairwise distance matrix was generated using Euclidean distance and multidimensional scaling was used to display the distribution of these 48 trees showing stool (red) and saliva (red) samples do not overlap; In Figure (b), the MLE tree for the tree samples combined is illustrated; and in Figures (c) and (d), the MLE tree for trees from stool and saliva samples are shown, respectively.

### Probabilistic Model

A unimodal probability model for graph-valued random objects has been derived and applied previously to several types of graphs (cluster trees, digraphs, and classification and regression trees) [30] and [27]. In this paper the model is applied to HMP trees constructed from RDP data as described above. Let 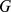 be the finite set of taxonomic trees with elements 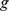, and 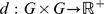
an arbitrary metric of distance on 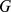. We have the probability measure H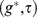 defined by
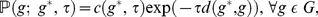
(1)where 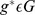 is the modal or central tree, 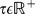
is a concentration parameter, and 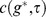 is the normalization constant. The probability model in (1) is known as the Gibbs distribution, the distribution that maximized the entropy providing the greatest sampling diversity [29]. Note that if 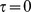, 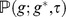 becomes a uniform distribution on 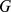, and if 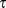 is large then the trees are concentrated around 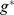, in which case the data provides information about the central tree.

**Table 3:** P-values of two sample test comparison using LRT statistics and 1000 bootstraps on HMP data formed by 24 subjects, region V3–V5.

p-value	anterior-nares	attached-gingivae	buccal-mucosa	hard-palate	l-retroauricular-crease	mid-vagina	palatine-tonsils	posterior-fornix	r-retroauricular-crease	saliva	stool	subgingival-plaque	supragingival-plaque	throat	tongue-dorsum	vaginal-introitus
anterior-nares	1															
attached-gingivae	*	1														
buccal-mucosa	*	0.10	1													
hard-palate	*	0.04	0.07	1												
l-retroauricular-crease	*	*	*	*	1											
mid-vagina	*	*	*	*	*	1										
palatine-tonsils	*	0.01	*	0.27	*	*	1									
posterior-fornix	*	*	*	*	*	0.60	*	1								
r-retroauricular-crease	*	*	*	*	0.46	*	*	*	1							
saliva	*	*	*	*	*	*	*	*	*	1						
stool	*	*	*	*	*	*	*	*	*	*	1					
subgingival-plaque	*	*	*	*	*	*	*	*	*	*	*	1				
supragingival-plaque	*	*	*	*	*	*	*	*	*	*	*	0.05	1			
throat	*	*	*	0.01	*	*	0.09	*	*	*	*	*	*	1		
tongue-dorsum	*	*	*	*	*	*	*	*	*	*	*	*	*	0.01	1	
vaginal-introitus	*	*	*	*	*	0.47	*	0.11	*	*	*	*	*	*	*	1

P-values are rounded off to two decimal places and * denotes P-values <0.001.

#### Distance metric

Two broad strategies exist for defining a suitable distance metric 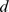 in tree space [35], [30], [36]: one approach focuses on counting the number of times we prune a branch (edge) or add a branch (edge) to transform one tree into another, and the second approach focuses on mapping trees into alternative mathematical structures for which natural metrics already exist. In this work we will focus on the latter approach, more specifically, we will focus on mapping trees into normed spaces.

In general, any finite graph defined on a set of labeled vertices or nodes can be uniquely characterized by mapping it into the space of matrices through the vertex-adjacency matrix 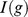 where 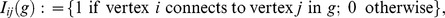 and for a weighted graph it can be simply defined as 
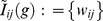
 where 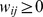 is the edge weight linking vertices *i* and *j*. If 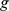 is an undirected graph its vertex-adjacency matrix is symmetric. The distance metric 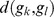 is given by the Frobenius norm [37] of the difference between the vertex-adjacency matrices of 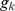 and 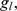 i.e. 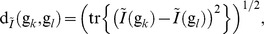 where 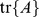 is the trace of the matrix 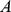. In the case of the RDP trees, the vertices are taxa labeled according to a Linnaean-taxonomic classification. Therefore not every pair of vertices is connected by an edge, for example, Bacilli is a descendent of Firmicutes but not of Bacteroidetes. This implies that 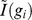 is a sparse matrix with many of its elements being always zero for any RDP tree sample. Moreover, the diagonal elements of 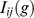 and 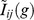 are always equal to zero since the graphs which concern our work are simple graphs which do not have loops. The above observations led us to consider a more efficient representation of RDP trees by mapping the weights of the edges that do exist, according to the Linnaean-taxonomic classification, into a vertex-adjacency vector 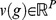 where 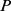 is the total number of existing edges. The distance metric 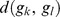 is the Euclidean norm of the difference between their adjacency-vectors and it is given by 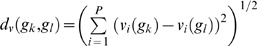. Mapping trees to Euclidean space facilitates the analysis and visualization of Tree objects, and the fitting of any probability model is computationally simpler in this space. However, one of the drawbacks of this metric is that it weight branches with lower range of values (e.g., genus level) do not contribute to differentiate one tree from another. Note that it is easy to show that for simple graphs metrics 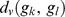 and 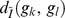
are related as follows: 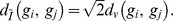 Also, if all the weights are considered to be 0 or 1, then 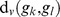 is equivalent to the square root of the Hamming distance between the trees, which is the number of edges discrepancies between two graphs. In [29] the authors show that under this metric the normalization constant is a function of 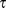 only, namely, it is independent of the central graph 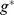.

#### Normalization constant

The space of RDP trees is continuous and constrained. In fact, by construction of the RDP tree the edge weights (the sum of confidence levels) are monotonically decreasing as we travel from the root, the vertex at the kingdom level, to the leaves, the vertices at the genus level. This means that 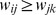, where 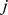 is the common taxonomical level, 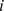 is its respective and unique parent node of 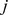, and 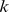 denotes any of the descendant nodes of 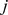. Moreover, all weighted edges are nonnegative. Therefore, since a weighted edge 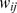 is an element of the vertex-adjacency vector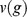, we have that for any RDP tree 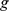 the following vector inequality should be satisfied: 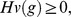 where 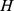 is a 0, 1, −1 matrix describing the set of inequalities of the type 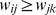 and 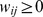. Hence, the normalization constant 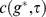 can be computed using the vertex-adjacency vector mapping as follows:
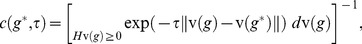
(2)where the integral is defined on the subspace 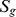 formed by the set of inequalities 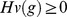. It is straightforward to show that a lower limit on 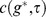 is given by,



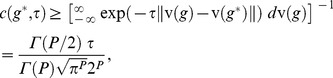
(3)where 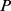 is the dimension of 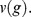 The lower limit depends only on 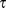 and on 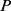. Note that since the exponential function in 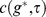 is symmetric around 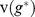, then 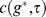 would tend to the lower limit as 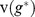 moves away from the boundaries of 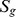
(including the origin). For a given 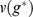, the difference between 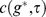 and the lower limit depends on the concentration parameter 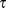, i.e., the larger its value the smaller the difference.

### Model Parameter Estimation

To estimate 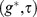 from a set of sample trees we use a maximum likelihood estimate (MLE) approach. In particular, for a random sample of 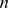 observed trees, 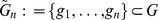, the log-likelihood is given by
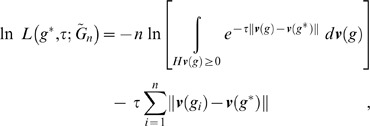
(4)and the MLE 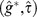 are such that 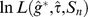 is maximum, 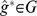, and 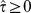. Note that Banks and Constantine [30] pointed out, the fact that the likelihood equation (4) contains two terms whose importance depends on the value of 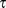. If 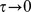 then (4) is dominated by the first term of the equation, and the points (trees) becomes less important. In this case the likelihood is a non-linear function of the distances between the trees in a neighborhood around 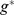. However, for large 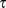, the second term dominates the likelihood, and the data points (trees) are of primary importance. In this case, the likelihood is a linear combination of the distances between the observed trees and the current estimate of 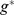. Following the analysis of Banks and Constantine [30], it can be shown that the MLE 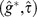
must satisfy the following equation:



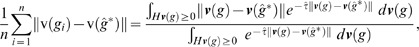
(5)where



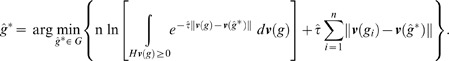
(6)


Solving the above equations for 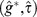 is hard, and thus it requires numerical search algorithm to obtain an approximation solution. Shannon and Banks in [27] developed an iterative algorithm to compute an approximate MLEs 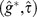 using equations (5) and (6), however their approach is specific to the set of unweighted trees (classification trees) and to the distance metric based on a weighted sum of the number of discrepant path of a certain length across all possible path lengths (See [27] for more details.) An algorithm considering the normalization constant would imply solving a multidimensional integral of an exponential function defined on the whole tree space, which does not have a closed-form solution. A Monte-Carlo integration approach incorporating Importance Sampling [38] could be attempted however at the cost of introducing a high computational complexity. Here, instead, we will approximate the likelihood function in (4) by replacing the normalization constant with its lower limit as given in (3), and thus the approximate MLEs 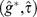 are given by,
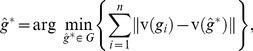
(7)

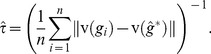
(8)


Note that solving the minimization problem in (7) with respect to 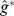 is equivalent to solving it with respect to the vertex-adjacency vector 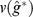 since 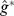 is uniquely characterized by 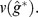 The unconstrained minimization problem with respect to 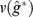 is also known as the Fermat-Weber location problem [39], and its solution is given by the geometric median of the vertex-adjacency vectors 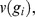 for 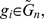 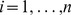. If the set of 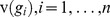 are not collinear, the expression 
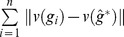
 is strictly convex and hence there is a unique minimum, 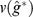. If 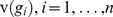 are collinear, the minimum is given by the dimension-wise median and hence it may not be unique [39]. There are no analytic solutions to compute the geometric median. Here we use the Weiszfeld iterative algorithm (see [39] for more details) with initial solution given by the mean tree, i.e., the average of the set of adjacency-vectors 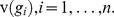 Though the proposed MLE approximation is computationally attractive, improving upon them is an interesting problem for future research.

### Two-sample Test Comparison

We are interested in assessing whether the distributions 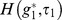 and 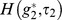 from two metagenomic populations are the same or different, which is equivalent to evaluating whether their respective parameters are the same or different. The corresponding hypothesis is given as follows:
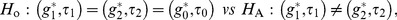
(9)where 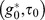 is the common parameter vector. Since the parameters under both hypothesis are unknown, we use the likelihood-ratio test (LRT) to evaluate (9), which is given by,



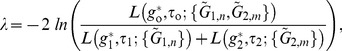
(10)where 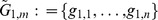 and 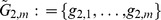 are the sets containing n and m random samples of trees from each metagenomic population, respectively. We assume that the model parameters are unknown under both the null and alternative hypothesis, therefore, we estimate these using the ML procedure described in Section 4, and compute the corresponding p-value using non-parametric bootstrap (see [40] and [29] for more details.)

## Results

### Application to Human Microbiome Data

We apply the HMP taxonomic tree OODA methods developed here to existing HMP data formed by 24 subjects (see Methods Section, under HMP Data Description and Data Structure, and reference there in for a complete description). An R-package has been developed containing the implementations of the visualization and methods proposed above [32]. In all our analyses below we only selected samples that have more than 1000 reads and, as a consequence, we excluded the right and left antecubital fossa sites since these ended up having 3 and 4 samples each.

For a given set of HMP stool samples we show in Figure 2 a multidimensional scaling (MDS) plot based on the pairwise Euclidean distance of all taxonomic trees. The MDS plot shows the distribution of the taxonomic trees in 2 dimensions. Individual taxonomic trees are shown around the MDS plot to illustrate how the tree structure varies. The trees are displayed using a circular graphical representation in which the root of the tree is at the center and same taxonomic-level nodes are placed at a fix radius around the center at a fix order, allowing each taxonomic lineage to be represented in a fixed consistent position in each tree. In this plots only genera are listed around the circumference of the plot. The tree branches are color coded to represent different ranges of weight values (see color table at the bottom left side of the figure). Blue denotes the branches with the highest confidence among all, and red denote the branches with lowest confidence. The maximum likelihood estimation, 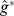, summarizes the tree distribution, and is the tree structure (microbiota) that maximizes the likelihood of seeing the data we observed. In Figure 3 we illustrate the corresponding MLE tree for the saliva samples illustrated in Figure 2.

To illustrate differences within a body site but across variable regions of the 16S rRNA gene, stool samples for 24 subjects were sequenced at variable regions V1–V3 and V3–V5, mapped to the RDP database, and a taxonomic tree estimated for each sample. Figure 4A shows a multidimensional scaling used to display the distribution of these 48 trees showing V1–V3 (blue) and V3–V5 (red) samples are overlapping; Figure 4B shows the MLE tree estimated after combining the two groups and Figure 4C and Figure 4D illustrates the MLE trees for samples corresponding to V1–V3 and V3–V5 regions, respectively. The LRT test of the null hypothesis that the microbiota distributions in variable regions V1–V3 and V3–V5 are the same is not rejected with p-value = 0.26, based on 1000 bootstraps, and is confirmed visually by the similarities in the MLE trees for these two regions. (Note that the structure of these trees places the same bacteria taxon at the same location on the tree, so visual branching comparisons are valid.) We conclude from this analysis that the central trees are the same for V1–V3 and V3–V5 and the combined MLE tree should be used as the best estimate of the central tree of the stool samples. Table 2 shows the p-values of the LRT statistics applied to each body site to test for similarities between samples from regions V1–V3 and V3–V5. In general, all of the sites except for attached-gingivae, buccal-mucosa, palatine-tonsils, and saliva, did not reject the null hypothesis that the distributions of samples from regions V1–V3 and V3–V5 are same based on a statistical significance of 5%.

To illustrate differences across body habitats, stool and saliva samples for 24 subjects were sequenced, mapped to the RDP database, and a taxonomic tree estimated for each sample. In Figure 5A we computed the Euclidean pairwise distance matrix between the trees (region V3–V5) and multidimensional scaling was used to display the distribution of these 48 trees showing stool (red) and saliva (blue) samples are visually distinct. Figure 5B displays the MLE tree estimated for the two body site groups combined, and the MLE trees estimated for stool and saliva separately are shown in Figure 5C and 5D, respectively. The LRT test of the null hypothesis that the distribution parameter in stool and saliva are the same is rejected with p-value < 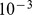, based on 1000 bootstraps, and is confirmed visually by the differences in the MLE trees for these two body habitats. We conclude from this analysis that the distributions are different for the two groups, and the MLE trees fit separately to the body habitats are the best estimates of their corresponding central trees.

In Table 3 we show the p-values of the LRT statistics applied to all possible pairwise comparisons of body habitats. It can be seen that, based on a statistical significance of 5%, the LRT statistic rejects the null hypothesis that the distribution parameters are same on pair of samples from body habitats located on anatomical regions physically separated, while in the case of some body habitats sharing the same anatomical region, e.g., vaginal sites, the null hypothesis is accepted. It is important to emphasize that we cannot conclude that the MLE trees are different using the p-values of the LRT statistics (only similarities can be concluded) since this test statistic assess for changes in both the central mode tree 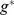 and the dispersion parameter 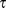 simultaneously. Note that multiple hypothesis testing correction can be performed here, however, the example is for illustration purposes only and so we did not emphasize the biological implication of our results.

In addition, for purpose of comparison, we apply Analysis of Similarity (ANOSIM) [25], which is a multivariate and non-parametric test widely used in community ecology to compare the variation in taxa abundance distribution among samples from different groups or treatments. ANOSIM operates directly on any measures of dissimilarity between the samples. Here, we compare habitat sites using the Bray-Curtis distance [41] between the taxa abundance information among samples at the genus level. We found that the results the ANOSIM test in all pairwise comparisons between habitat sites was consistent with the results provided by our method (Table 3). For example, the ANOSIM comparison of the saliva and stool samples shown in Figure 5 resulted in a statistically significant difference (P = 0.001) between the 2 groups, which is consistent with what would be expected from a visual examination of the separation of the two groups using MDS based on Bray-Curtis distance, and consistent with the taxonomic tree comparison (P< 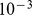).

## Discussion

We propose a novel parametric statistical inference method for analyzing HMP data which is naturally represented in the form of a taxonomic tree. Using methods from Object Oriented Data Analysis (OODA), we applied classical statistical methods for inference and hypothesis testing to the analysis of HMP RDP data. In particular, we applied a unimodal probability model which depends on a dispersion parameter and central mode tree. We introduce an approximate MLE procedure for estimating model parameters and we derive LRT statistics for comparing the distributions of two metagenomic populations.

Within the framework of representing HMP data by taxonomic trees there are currently two basic approaches for defining (estimating) the core: First, a consensus tree can be built by combining common branches from the samples and removing unusual samples, i.e., the intersection tree. This approach defines the core as the set of organisms that are present in a particular body site in all or in a vast majority of individuals [42]. However, this graph-theoretic approach would eliminate taxa that are alternate sources of the same biological function and thus would each be present in some but not all tree samples. Moreover, it assumes that every tree sample is correct and no estimates of error or methods for inference are available; and second, all sequence reads from each sample can be combined and a single taxonomic tree constructed, i.e., the union tree. This approach will likely produce a biased estimate since rare sequence matches and spurious matches caused by error in sequencing or incorrect matching to taxa with conserved genetic regions will be retained. Therefore, the MLE tree, 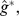 proposed in this work, stands as a more appropriate definition of core since it corresponds to the microbiome most likely to be observed, and is “core” in that sense. The great comparative advantage of using 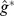 as a core definition is that by using a probabilistic model it deals with the variability of organisms present in the samples across subjects avoiding a highly constrained and deterministic definition of the core. Note that the approximate MLE tree tends to incorporate more branches that any sample tree since the MLE tree correspond to the geometric median of all the samples, and thus it is the results of a linear combination of all sample tree. After reviewing this point more carefully, we realized that the MLE tree has the properties of a supertree, potentially a much larger tree than some or all of the original tree data. Again, we believe this is an important component of the definition of the core microbiome. In a future work we will study the biological insights that the MLE tree can provides as a core when analyzing metagenomic samples of specific habitat sites.

Our approach is based on the assumption that a unimodal model fits the set of tree samples, which might not always be valid [43]. Goodness-of-fit test for the unimodal model applied to binary trees has been discussed in [29] and [30]. However, the Pearson Chi-Square approach proposed in [29] applies only to the set binary trees which are discrete objects and unweighted, thus not applicable to the tree objects of our paper. To the best of our knowledge goodness-of-fit test statistics for this model applied on weighted trees has not be derived yet. We are currently working on deriving more general models such as finite mixture model of the unimodal probability model to assess for the existence of several modes in the data, e.g., due to the presence of subgroups of trees within the data that correspond to sample subgroups. The estimation of the corresponding core microbiomes of each subgroup can be obtained by using the conventional EM algorithm combined with the MLE search algorithm proposed in this work. Though, this is formally a modeling selection approach, it will provide a sense of how well the unimodal assumption holds in the data compared with multimodal alternatives. In addition, we will investigate several others distance measures between taxonomic tree objects, and assess how these metrics influence the estimation of the core microbiome and their impact on the performance of likelihood ratio tests.

The application of the LRT statistics to real HMP data formed by 24 subjects allowed testing for differences of core microbiomes across body habitats and variable regions within the same body site. When comparing the results of the LRT test with those obtained by ANOSIM [25], consistent conclusions were obtained. Though technically both methods assess different hypothesis about the samples, several advantages can be listed in favor of our approach when compared with ANOSIM. First, our methodology provided with an estimate of the central taxonomic trees for both body sites while ANOSIM does not provide a direct estimate of the average microbiome. Second, taxonomic analysis retains more information than ANOSIM which likely provides more power for the analysis. ANOSIM, like many existing metagenomic analysis methods, first reduces the data to a pairwise distance matrix which by definition contains less information. By this we mean that the distance between two samples does not indicate what the microbial composition is of those samples, or allows the average composition to be determined. These results illustrate the potential that our method has in guiding the analysis of the vast amount of HMP data is currently being generated (samples from 300 subjects, 18/15 body habitats, multiple visits, and multiple sequencing platforms), and in helping to bridge the transition from HMP technology development to clinical applications.
